# TRPV6-Mediated Ca^2+^ Signaling in Pancreatic Ductal Adenocarcinoma: From Cellular Physiology to Therapeutic Opportunities

**DOI:** 10.3390/cancers18101670

**Published:** 2026-05-21

**Authors:** Boshra Yosef, Viktória Venglovecz

**Affiliations:** 1 Department of Pharmacology and Pharmacotherapy, University of Szeged, 6720 Szeged, Hungary; boshra.yosef@med.u-szeged.hu; 2 Translational Pancreatology Research Group, Interdisciplinary Center of Excellence for Research Development and Innovation, University of Szeged, 6720 Szeged, Hungary; 3 Institute for Translational Medicine, Medical School, University of Pécs, 7624 Pécs, Hungary

**Keywords:** pancreatic ductal adenocarcinoma (PDAC), TRPV6, Ca^2+^ signaling, ion channels

## Abstract

Pancreatic ductal adenocarcinoma (PDAC) is a highly aggressive cancer type in which therapeutic options are limited, and the disease is characterized by a poor prognosis. In the development of pancreatic cancer, dysregulated Ca^2+^ signaling plays a key role by regulating proliferation, survival, metabolic adaptation, and tumor–microenvironment interactions. Among the Ca^2+^ channels, TRPV6 has emerged as a key regulator since this channel is highly selective for Ca^2+^ and frequently overexpressed in different types of cancers. The aim of this review is to summarize our current knowledge on the structure, regulation, and function of TRPV6, with emphasis on its cell-type specific roles within the pancreas. We describe the mechanisms by which TRPV6-mediated Ca^2+^ influx activates oncogenic signaling pathways, such as NFAT, AKT/mTOR, and NF-κB, and how this channel plays a role in intra- and extracellular pH regulation. In addition, the clinical relevance and potential contribution of TRPV6 to therapy resistance are discussed. Finally, we review pharmacological strategies and future perspectives regarding TRPV6 in PDAC.

## Simple Summary

Pancreatic cancer (PC) is one of the deadliest cancers, largely because it is often detected late and responds poorly to current treatments. Understanding the biological processes that drive this disease is essential for developing better therapies. One important factor is how cells handle Ca^2+^, a key signal that controls cell growth and survival. This review focuses on a specific Ca^2+^ channel, TRPV6, which is often expressed at higher levels in PC cells. We summarize what is currently known about how TRPV6 works in normal pancreatic cells and how its altered activity may contribute to cancer development and progression. We also discuss whether targeting this channel could offer new treatment options. Overall, this work highlights TRPV6 as a promising area for future research and potential therapeutic intervention in PC.

## 1. Introduction

The pancreatic ductal system has a pivotal role in the maintenance of the exocrine homeostasis by regulating HCO_3_^−^ secretion, ion transport, and luminal pH [1]. The normal function of these processes is highly dependent on tightly regulated Ca^2+^ signaling, which coordinates fluid secretion and ensures proper enzymatic activity in the duodenum. Since pancreatic ductal epithelial cells (PDECs) not only provide a structural but also a functional basis for the pancreas, alterations in their intracellular signaling pathways, especially those relating to Ca^2+^ signaling, can potentially contribute to disease development. Pancreatic ductal adenocarcinoma (PDAC) is one of the most frequent forms of pancreatic cancer (PC). PDAC is responsible for over 90% of PC cases [2]. The 5-year survival rate is less than 10%, mainly due to late diagnosis, rapid progression, and poor response to standard treatments, such as chemotherapy, surgery, and radiation [2]. In most cases, PDAC develops in the head of the pancreas, where it is associated with symptoms such as jaundice or abdominal pain that could allow earlier detection of the disease. In contrast, tumors in the body and tail often have worse outcomes. Therefore, understanding early molecular changes in PDECs is crucial in order to improve early detection and identify new therapeutic targets [3,4,5,6].

Ca^2+^ homeostasis plays a central role in PC biology because it is essential for normal exocrine secretion, ion transport, and pH regulation. When this balance is disrupted, sustained intracellular Ca^2+^ overload can cause mitochondrial injury as well as ATP depletion, and drive inflammatory or degenerative cascades [7,8]. However, the same disturbed Ca^2+^ signaling becomes advantageous for tumor cells, as it can promote survival, proliferation, and invasiveness, particularly when channels are upregulated. Therefore, in order to understand pancreatic carcinogenesis, it is fundamentally important to know the mechanisms that regulate Ca^2+^ homeostasis [9,10]. Transient receptor potential (TRP) channels are a diverse family of ion channels that mediate the transport of Ca^2+^ and other cations [11]. Their activity is highly involved in various cancer-related processes, such as cell proliferation, migration, invasion, metastasis, angiogenesis, and the development of chemotherapy resistance [12]. Several TRP subfamilies (TRPV, TRPC, TRPM, TRPA) have been investigated in different tumor types, and their selective modulation has emerged as a promising therapeutic strategy [12,13,14]. Understanding the molecular mechanisms of these channels is therefore crucial for developing targeted interventions that may limit tumor progression and improve patient outcomes.

TRPV6 is a highly Ca^2+^-selective member of the transient receptor potential vanilloid (TRPV) subfamily and plays a central role in epithelial Ca^2+^ transport [15]. TRPV6 is frequently overexpressed in several malignancies, including prostate, breast, ovarian, and colorectal cancers, where the elevated expression of the channel often associates with increased tumor aggressiveness and poor clinical outcome, underscoring its value as a potential therapeutic target [16,17,18,19]. Cryo-electron microscopy studies have described the regulation, gating properties, and structure of TRPV6, which provide a good basis for the development of channel-specific inhibitors [20,21]. Recent studies indicate that TRPV6 is also present in PDECs and that its expression significantly increases in PDAC compared with healthy pancreas tissue [22]. Functional studies suggest that TRPV6 enhances the proliferation, migration, and survival of PC cells through mechanisms driven by altered Ca^2+^ signaling [22]. Additionally, it has also been shown that genetic variants of TRPV6 strongly correlate with chronic pancreatitis development, indicating a possible mechanistic link between Ca^2+^ dysregulation, chronic inflammation, and pancreatic carcinogenesis [23,24,25]. Despite the accumulating data regarding TRPV6 in pancreatic pathology, no comprehensive review has summarized its expression patterns, molecular functions, and therapeutic relevance specifically in the context of PDECs. Therefore, the aim of this review is to integrate current knowledge on TRPV6 in pancreatic physiology and tumorigenesis, identify key gaps in understanding and evaluate its potential as a biomarker and therapeutic target in PDAC.

## 2. Molecular Structure, Regulation, and Function of TRPV6

**Figure 1:**
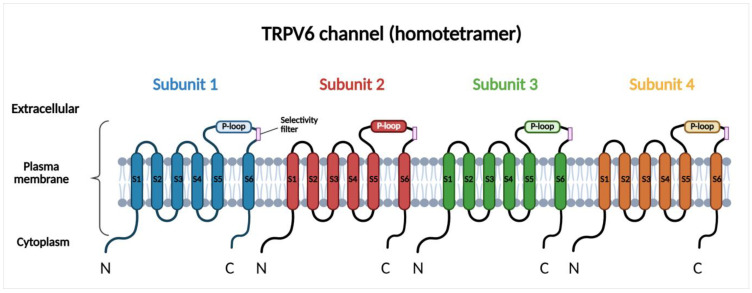
Structure of TRPV6. Schematic figure of the TRPV6 channel showing its homotetrameric structure within the cell membrane. Each subunit contains six transmembrane segments and a pore region that is highly selective for Ca^2+^. N: N-terminus; C: C-terminus; P-loop: pore loop. This figure was created in BioRender. Venglovecz V. (2026) https://app.biorender.com/illustrations/69f856634313385bd35fb801 (accessed on 15 May 2026).

TRPV6 is a Ca^2+^ selective channel localized to the plasma membrane, where it mediates constitutive Ca^2+^ influx. Under physiological conditions, TRPV6 is predominantly expressed in epithelial tissues involved in Ca^2+^ absorption and transport, including the small intestine, kidney, placenta, and exocrine glands, where it contributes to transcellular Ca^2+^ uptake and systemic Ca^2+^ homeostasis [15,26,27,28]. However, emerging evidence suggests that TRPV6 is also present in the membranes of intracellular vesicles, ref. [29], although its role is less well characterized and requires further investigation. TRPV6 is a homotetrameric transmembrane protein, with each subunit comprising six transmembrane segments (S1–S6) and a pore region. The pore region is located between the fifth and sixth segments and is responsible for the Ca^2+^ selectivity of the channel [26,27] (Figure 1). This region includes a re-entrant pore loop (P-loop) between S5 and S6 that extends into the central ion-conducting pore. The Ca^2+^ selectivity filter is formed by conserved negatively charged aspartate residues within the P-loops of all four subunits, which coordinate Ca^2+^ ions and confer high Ca^2+^ selectivity to the channel. High-resolution cryo-electron microscopy studies have described the molecular structure of TRPV6 and identified specific determinants that regulate ion permeation, gating, and inactivation [20,21]. The activity of the channel is highly dependent on the intracellular Ca^2+^ concentration. One of the major regulators of TRPV6 is calmodulin (CaM). CaM is a widely expressed Ca^2+^-binding messenger protein that senses changes in intracellular Ca^2+^ levels and transduces these signals by interacting with a wide range of target proteins. When intracellular Ca^2+^ increases, the excess Ca^2+^ binds to CaM, then the Ca^2+^-CaM complex interacts with the C-terminal regulatory domain of TRPV6 and inactivates the channel. This mechanism inhibits excessive Ca^2+^ entry and protects the cells from Ca^2+^ overload [30]. This regulatory mechanism ensures that TRPV6 is active at low intracellular Ca^2+^ concentrations while becoming inactive in response to elevations in cytosolic Ca^2+^. In contrast to voltage-gated Ca^2+^ channels, membrane depolarization does not affect the activity of TRPV6; therefore, this channel mainly contributes to basal and sustained Ca^2+^ entry. TRPV6 expression is also regulated at the transcriptional level. Vitamin D is one of the transcriptional regulators of TRPV6 that upregulates its expression, thereby enhancing Ca^2+^ influx and downstream Ca^2+^-dependent signaling [31,32]. Downstream, TRPV6-mediated Ca^2+^ signaling modulates Ca^2+^-dependent pathways, such as CaM-dependent kinases or calcineurin-NFAT (nuclear factor of activated T cells) signaling, thereby influencing gene transcription, cell proliferation, and survival [33]. Since the major role of TRPV6 in epithelial cells is to support physiological Ca^2+^ uptake and maintain normal Ca^2+^ homeostasis, ref. [34], dysregulated TRPV6 expression or activity leads to disruption of intracellular Ca^2+^ balance that promotes pathological signaling [35].

## 3. TRPV6-Mediated Ca^2+^ Homeostasis in Pancreatic Physiology

### 3.1. Pancreatic Ductal Cells

In the normal pancreas, the presence of TRPV6 in ductal cells has not been clearly demonstrated. Large-scale transcriptomic datasets of the human pancreas, including single-cell RNA-sequencing resources, show that TRPV6 transcripts are detectable within the exocrine pancreas; however, these studies did not specifically examine the presence of TRPV6 in ductal cells [36]. In addition, there are no data regarding the functional characterization of TRPV6 in ductal cells under physiological conditions. In contrast, several genetic and pathophysiological studies indirectly support the role of TRPV6 in ductal cells. Loss-of-function variants of TRPV6 induce altered pancreatic Ca^2+^ signaling and increase the risk of chronic pancreatitis, a disease in which dysregulated ductal Ca^2+^ homeostasis is one of the key pathological factors [24,37]. Based on this, it is hypothesized that TRPV6 participates in the regulation of luminal Ca^2+^ levels by promoting the uptake of Ca^2+^ from ductal fluid and thereby decreasing intra-ductal Ca^2+^ levels, calcification, and epithelial injury. In addition, TRPV6 expression has been detected and shown to be upregulated in human PDAC tissues and cell lines, and xenograft mouse models [22,38], supporting the notion that its expression may be more prominent under pathological than physiological conditions in the ductal cells.

### 3.2. Pancreatic Acinar Cells

In contrast to ductal cells, more data are available regarding the expression of TRPV6 in acinar cells. Recent studies using RNAscope and immunofluorescence approaches in mouse models of acute pancreatitis and freshly isolated mouse pancreatic acinar cells have demonstrated TRPV6 expression, with predominant localization at the plasma membrane [39]. In addition, it has also been shown, using patch-clamp techniques and the specific TRPV6 inhibitor SOR-C27, that the channel is functionally active. The TRPV6-mediated Ca^2+^ influx may contribute to intracellular Ca^2+^ replenishment during increased secretory activity, but can also generate pathological Ca^2+^ overload under injurious conditions [39]. Studies in experimental models of acute pancreatitis suggest that TRPV6-associated Ca^2+^ influx markedly amplifies cytosolic Ca^2+^ elevations, which lead to mitochondrial dysfunction, premature activation of digestive enzymes, and trigger inflammatory cascades that are characteristic features of acinar cell injury [39]. In addition, genetic studies in human cohorts have shown that loss-of-function variants of TRPV6 increase susceptibility to pediatric-onset pancreatitis, particularly when they coexist with other pancreatitis-associated susceptibility genes [23].

### 3.3. Endocrine Pancreas

Transcriptomic analyses of human pancreatic islets have shown TRPV6 expression in both β and α cell populations [40]. In addition, increasing evidence from in vitro cell-based studies indicates that TRPV6 contributes to cell proliferation and gene transcription in β-cells rather than acute insulin secretion [41]. The functional role of TRPV6 in intracellular Ca^2+^ regulation was directly characterized in the insulin-secreting rat INS-1E β-cell line [41]. Downregulation of TRPV6 caused a marked reduction in Ca^2+^ influx that led to decreased β-cell proliferation and reduced insulin mRNA expression. It has also been shown that the TRPV6-mediated Ca^2+^ entry activates the calcineurin–NFAT signaling pathway, a key regulator of β-cell growth and gene transcription. Importantly, TRPV6 downregulation did not affect glucose-stimulated insulin exocytosis, indicating that voltage-gated Ca^2+^ channels remain the main mediators of stimulus–secretion coupling in pancreatic β-cells. Consistent with these observations, studies on TRPV channel function in pancreatic β-cells indicate that TRP-mediated Ca^2+^ uptake is more likely to contribute to the regulation of intracellular Ca^2+^ homeostasis than to triggering rapid insulin secretion [42]. The growth-regulatory role of TRPV6 in the endocrine pancreas is further supported by studies in the human pancreatic neuroendocrine BON-1 cell line, where TRPV6-mediated Ca^2+^ influx regulates cell proliferation through a Ca^2+^-dependent mechanism involving activation of NFAT signaling [43]. Although these findings provide important mechanistic insights regarding the role of TRPV6 in the endocrine pancreas, the precise physiological relevance of TRPV6 in normal β-cells remains incompletely understood and requires further investigation. The presence and the physiological and pathophysiological significance of the TRPV6 channel in the exocrine and endocrine pancreas are summarized in Table 1.

## 4. TRPV6-Mediated Ca^2+^ Signaling in Pancreatic Cancer

**Figure 2:**
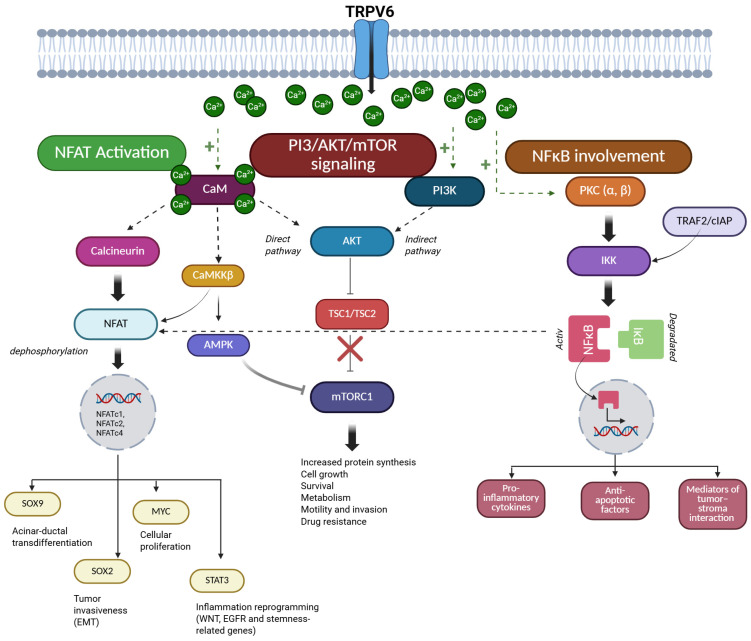
Hypothetical model of TRPV6-mediated Ca^2+^-dependent signaling pathways in pancreatic ductal cells. Ca^2+^ influx through TRPV6 activates multiple downstream signaling pathways in pancreatic ductal adenocarcinoma (PDAC). Ca^2+^-calmodulin (CaM) activates calcineurin, leading to NFAT dephosphorylation and transcription of genes associated with acinar-to-ductal trans-differentiation (SOX9), proliferation (MYC), tumor invasiveness (SOX2), and inflammatory reprogramming (STAT3). In parallel, Ca^2+^ signaling contributes to AKT activation through both direct CaM-dependent mechanisms and indirect PI3K-mediated pathways. Activated AKT inhibits the TSC1/TSC2 complex, resulting in mTORC1 activation, which supports protein synthesis, cell growth, survival, metabolic adaptation, invasion, and drug resistance. Additionally, Ca^2+^-dependent activation of PKC contributes to IKK-mediated phosphorylation and degradation of IκB, enabling NF-κB nuclear translocation and transcription of genes involved in inflammation, anti-apoptotic signaling, and tumor–stroma interactions. AKT: protein kinase B; AMPK: AMP-activated protein kinase; CaM: calmodulin; CaMKKβ: Ca^2+^/calmodulin-dependent protein kinase kinase beta; cIAP: cellular inhibitor of apoptosis protein; IKK: IκB kinase complex; IκB: inhibitor of κB; mTORC1: mechanistic target of rapamycin complex 1; MYC: MYC proto-oncogene; NFAT: nuclear factor of activated T cells; NF-κB: nuclear factor kappa-light-chain-enhancer of activated B cells; PI3K: phosphoinositide 3-kinase; PKC: protein kinase C; SOX2: SRY-box transcription factor 2; SOX9: SRY-box transcription factor 9; STAT3: signal transducer and activator of transcription 3; TRAF2: TNF receptor-associated factor 2; TRPV6: transient receptor potential vanilloid 6; TSC1/TSC2: tuberous sclerosis complex 1/2. The red cross indicates inhibition of TSC1/TSC2-mediated suppression of mTORC1 by AKT, while the gray inhibitory line represents AMPK-mediated negative regulation of mTORC1. This figure was created in BioRender. Venglovecz V. (2026) https://app.biorender.com/illustrations/69ca264ab48bae08c82e48bb (accessed on 9 May 2026).

PDAC is a highly aggressive malignancy where Ca^2+^ signaling plays a key role in the regulation of tumor behaviors such as growth, survival, and invasion. The effect of Ca^2+^ is not direct; it is mediated through signaling cascades, including NFAT, PI3K/AKT/mTOR, or NF-κB (Figure 2). Ca^2+^ signaling also regulates intra- and extracellular pH homeostasis, a critical determinant of PDAC cell survival within the acidic tumor microenvironment. The following sections will discuss how TRPV6-mediated Ca^2+^ influx coordinates these pathways to promote PDAC progression and therapy resistance.

### 4.1. TRPV6 and Ca^2+^-Dependent Oncogenic Signaling Pathways

#### 4.1.1. NFAT Activation via Ca^2+^ Entry

Multiple Ca^2+^ entry mechanisms exist in PDAC cells, such as the classic store-operated Ca^2+^ entry (SOCE) or TRP channels; however, persistently elevated intracellular Ca^2+^ levels—such as those supported by constitutive Ca^2+^ influx via TRPV6—represent the critical determinant of downstream signaling. Sustained elevations in intracellular Ca^2+^ levels activate the Ca^2+^/CaM-dependent protein phosphatase, calcineurin [44]. Calcineurin is a serine/threonine phosphatase. The main target of calcineurin is the nuclear translocation of NFAT transcription factors. NFAT is a family of Ca^2+^-regulated transcription factors that control gene expression in response to intracellular Ca^2+^ signals. Dephosphorylation of NFAT by calcineurin leads to the translocation of NFAT family members, such as NFATc1, NFATc2, and NFATc4 to the nucleus and initiates transcription of genes related to tumor growth, survival, and invasion [7]. In PDAC cells resistant to gemcitabine, a nucleoside analog chemotherapeutic agent widely used in PC treatments, increased STIM1 expression enhances SOCE, which leads to the activation of NFATc2 and drives epigenetic remodeling characterized by increased H3K27 acetylation at stress-responsive gene loci, thereby contributing to chemoresistance [45]. NFAT signaling promotes trans-differentiation of acinar cells into ductal cells by inducing the expression of the ductal transcription factor SRY-box 9 (SOX9). In addition, NFAT induces inflammatory reprogramming through functional cooperation between NFATc1 and signal transducer and activator of transcription 3 (STAT3). This interaction increases the transcription of genes associated with Wnt and EGFR signaling, as does the expression of stemness-related genes [44]. In parallel, NFAT activation facilitates cell-cycle progression by displacing SMAD3 repressor complexes from the c-Myc promoter, thereby increasing MYC expression and promoting cellular proliferation [46]. SMAD3 is a transcription factor and a key downstream effector of transforming growth factor-β (TGF-β) signaling, where it typically functions as a regulator of gene expression involved in cell-cycle control and growth inhibition. NFAT signaling also promotes tumor invasiveness through interaction with SOX2. This protein acts as a transcription factor and enhances the expression of epithelial–mesenchymal transition (EMT)-associated transcription factors, such as E-box-binding homeobox 1 and snail family transcriptional repressor 1, thereby promoting mesenchymal-like phenotypes and increased migratory potential [44]. In addition to the calcineurin-NFAT axis, Ca^2+^ influx in PDAC cells also induces parallel Ca^2+^-sensitive signaling mechanisms that reinforce NFAT-dependent transcriptional programs. Ca^2+^/CaM-dependent kinase kinase β (CaMKKβ) supports NFAT-dependent transcriptional activity, thereby amplifying Ca^2+^-driven proliferative signaling in PDAC cells [44]. The functional relevance of Ca^2+^-NFAT coupling in PDAC was also confirmed by pharmacological inhibition of Ca^2+^ release-activated Ca^2+^ (CRAC) channels. RP4010, a small molecule CRAC channel blocker, reduces the nuclear translocation of NFAT1 and suppresses tumor growth in patient-derived PDAC models, demonstrating that sustained Ca^2+^ influx is required to maintain oncogenic NFAT signaling and highlighting the therapeutic potential of disrupting Ca^2+^-NFAT communication [47].

#### 4.1.2. PI3K/AKT/mTOR Signaling Triggered by Ca^2+^

In PDAC, Ca^2+^ signaling is an important upstream regulator of the PI3K/AKT/mTOR pathway, which regulates cell growth, metabolism, survival, and therapeutic resistance. The PI3K/AKT/mTOR pathway is a central intracellular signaling cascade in which phosphoinositide 3-kinase (PI3K) generates lipid second messengers that initiate pathway activation. AKT (protein kinase B) functions as a serine/threonine kinase that promotes cell survival and growth, while mechanistic target of rapamycin (mTOR) acts as a key regulator of protein synthesis, metabolism, and cellular anabolic processes. Sustained elevations in intracellular Ca^2+^ facilitate AKT activation and couple extracellular stimuli to growth-promoting and pro-survival pathways [48,49,50]. These observations are supported by studies in human PDAC tissues demonstrating AKT and mTOR expression, as well as by experimental data derived from cancer cell lines and mechanistic studies in various cellular models. Ca^2+^-dependent activation of AKT occurs through both direct and indirect mechanisms. The direct pathway includes CaM, which is activated by increased intracellular Ca^2+^ levels. Activated CaM directly interacts with AKT and promotes its phosphorylation and kinase activity, thereby linking Ca^2+^ influx to AKT signaling [51,52]. In parallel, Ca^2+^ signaling modulates PI3K activity, thereby reinforcing pathway activation and sustaining oncogenic signaling outputs [53,54,55]. Ca^2+^-AKT interactions play a role in the development of resistance to apoptosis and support anabolic growth, particularly under conditions of metabolic or therapeutic stress [50,56].

Downstream of AKT, Ca^2+^-regulated signaling pathways converge on mTOR. AKT activates mTOR complex 1 (mTORC1) by inhibiting the tuberous sclerosis complex (TSC1/TSC2), which normally suppresses mTORC1 activity [56,57,58]. In addition, CaMKKβ provides an alternative route linking Ca^2+^ signals to mTOR regulation via the AMP-activated protein kinase (AMPK) [48,49,50]. AMPK acts as a cellular energy sensor that regulates metabolic homeostasis and inhibits mTOR signaling under energy stress. Through the AMPK-mTOR axis, fluctuations in intracellular Ca^2+^ can modulate mTOR activity, balancing anabolic growth with metabolic stress responses. In PDAC, AKT/mTOR activity supports multiple cancer-related processes. Enhanced mTOR signaling promotes increased protein translation, lipid biosynthesis, and mitochondrial function, thereby maintaining tumor growth in nutrient-poor or hypoxic microenvironments [48,50,51]. Ca^2+^-induced reinforcement of AKT/mTOR signaling has also been implicated in therapy resistance, including reduced sensitivity to gemcitabine, by enhancing cell survival pathways, autophagy-mediated stress tolerance, and metabolic flexibility [50,51,52,53]. These functional effects have been demonstrated in vitro in PC cell lines and are supported by in vivo and translational studies, including analyses of patient-derived samples [52,53,54]. Beyond its role in cell growth and survival, AKT/mTOR signaling also contributes to PDAC cell motility and invasion, promoting cytoskeletal remodeling and EMT-associated transcriptional programs, thereby facilitating local invasion and metastatic dissemination [48,54]. Experimental studies demonstrate that disruption of Ca^2+^ signaling can attenuate AKT/mTOR activity and reduce malignant phenotypes in PC models, highlighting the functional interplay between Ca^2+^ entry and PI3K/AKT/mTOR signaling [48,53]. These findings indicate that the AKT/mTOR pathway is a key downstream effector of dysregulated Ca^2+^ entry in PDAC. Inhibition of Ca^2+^ entry pathways or AKT/mTOR signaling therefore represents a potential strategy to disrupt this adaptive signaling network in PC.

#### 4.1.3. NF-κB Involvement in Ca^2+^-Regulated Processes

Increased intracellular Ca^2+^ levels also influence the activation of nuclear factor kappa-light-chain-enhancer of activated B cells (NF-κB). NF-κB is a family of transcription factors that regulate the expression of genes involved in inflammation, cell survival, and immune responses. The main mediators between Ca^2+^ signaling and NF-κB activation are protein kinases and adaptor complexes. Ca^2+^-dependent activation of protein kinase C (PKC) isoforms (PKCα and β) can modulate the IκB kinase (IKK) complex, which leads to the phosphorylation and degradation of IκB proteins, enabling nuclear translocation of NF-κB subunits [55,56]. IκB proteins act as cytoplasmic inhibitors of NF-κB, retaining it in an inactive state until phosphorylation-induced degradation occurs. In PDAC, activation of NF-κB plays a key role in tumor progression by promoting the expression of pro-inflammatory cytokines (e.g., IL-6, TNF-α), anti-apoptotic factors (Bcl-xL, XIAP), and mediators of tumor–stroma interaction [57,58,59]. NF-κB signaling in PDAC interacts with TNF receptor–associated adaptor proteins such as TRAF2 and cellular inhibitor of apoptosis proteins (cIAPs), which coordinate canonical and non-canonical NF-κB activation [56,60]. These adaptor complexes integrate inflammatory and stress signals and enhance NF-κB-dependent gene expression that supports PC cell survival. In addition, NF-κB can cooperate with NFAT, linking inflammatory signaling with Ca^2+^-dependent gene expression [59]. NF-κB signaling also promotes EMT-associated transcriptional programs and plays a role in the increased expression of matrix metalloproteinases and the development of invasive, stem-like phenotypes [61]. Importantly, dysregulated NF-κB signaling is closely linked to chemoresistance in PDAC. Persistent NF-κB activation enhances the expression of anti-apoptotic and stress response genes, thereby reducing sensitivity to cytotoxic agents such as gemcitabine [59,62]. Accordingly, experimental inhibition of upstream Ca^2+^-sensitive regulators attenuates NF-κB activity and restores drug sensitivity in PC models, underscoring the functional relevance of the Ca^2+^–NF-κB axis [63,64,65].

### 4.2. Role of TRPV6 in pH Regulation in Pancreatic Cancer

Maintenance of intra- and extracellular pH homeostasis plays a critical role in the survival, proliferation, and invasiveness of PDAC cells [66,67]. Typically, tumor cells create an acidic extracellular environment, mainly through metabolic reprogramming and active proton secretion, while maintaining an alkaline intracellular pH that favors proliferation and resistance to apoptosis [66,68]. Ca^2+^ signaling is an important coordinator of acid–base homeostasis, as fluctuations in intracellular Ca^2+^ affect the activity of ion transporters and metabolic enzymes [8,69,70]. In this context, TRPV6-mediated Ca^2+^ influx may function as an upstream contributor to pH regulation by sustaining Ca^2+^-dependent signaling pathways in PC cells. Although there is no direct evidence that TRPV6 influences the activity of ion transporters in PDAC cells, several Ca^2+^-dependent kinases and phosphatases have been shown to regulate the function of certain transporters, such as the Na^+^/H^+^ exchangers (particularly NHE1), vacuolar H^+^-ATPases (V-ATPases), and HCO_3_^−^ transport systems in cancer cells [71,72,73]. NHE1 activation, in particular, causes intracellular alkalinization and extracellular acidification in pancreatic and other solid tumors [74,75,76]. Sustained Ca^2+^ influx via TRPV6 could therefore reinforce signaling environments that favor proton extrusion and maintenance of alkaline cytosolic pH.

Ca^2+^ signaling also affects metabolic reprogramming, which directly influences tumor pH. Ca^2+^ uptake by mitochondria regulates oxidative metabolism, whereas hypoxia promotes glycolysis and increases lactate and proton production [77,78]. In PDAC, metabolic reprogramming and increased lactate production contribute to extracellular acidification, which promotes tumor aggressiveness [68,79,80]. Due to the sustained Ca^2+^ signaling, TRPV6 may indirectly support metabolic adaptations that intensify acid production and, as a result, the acidification of the extracellular environment. Altered intra- and extracellular pH also plays a role in the development of drug resistance. Acidic tumor microenvironments reduce the efficacy of weakly basic chemotherapeutic agents and activate stress-response pathways that enhance cell survival [67,80]. Persistent Ca^2+^ signaling can support cell survival under metabolic stress [81], suggesting that TRPV6-dependent Ca^2+^ entry could contribute to adaptive pH–metabolic responses that protect PDAC cells during therapy.

## 5. Clinical and Translational Relevance of TRPV6 in Pancreatic Ductal Adenocarcinoma

Several clinical and experimental studies suggest that TRPV6 contributes to the clinical behavior of PDAC. These studies have demonstrated that TRPV6 is overexpressed in PDAC tissues, and the expression levels of TRPV6 increase in parallel with tumor stage and histological grade. In addition, elevated TRPV6 expression correlates with higher Ki-67 proliferation indices and more aggressive tumor phenotypes, indicating that there is a strong correlation between TRPV6 expression levels and disease progression [22,38,82]. Importantly, high TRPV6 expression is associated with reduced overall survival and poorer disease outcomes, suggesting that TRPV6 may serve as an independent prognostic biomarker in PDAC [38]. Therefore, TRPV6 expression in tumor samples may support diagnostic stratification, particularly when used together with established pathological markers. Polyclonal antibodies such as rb79 have been developed for the accurate detection of TRPV6 in patient samples. This advancement supports their potential use as diagnostic markers or as indicators of tumor activity [83]. Besides its prognostic value, TRPV6 is also implicated in therapy response. Experimental studies show that knockdown of TRPV6 decreases proliferation, enhances apoptosis, and increases sensitivity to chemotherapeutic agents such as gemcitabine and 5-fluorouracil [22]. Pharmacological inhibition of TRPV6, including peptide antagonists such as SOR-C13, has shown safety in early-phase clinical trials and preliminary evidence of disease stabilization in advanced solid tumors [84]. TRPV6 may also act as a molecular link between chronic pancreatic inflammation and tumor progression. Loss-of-function variants of TRPV6 have been associated with chronic pancreatitis [23,37], a known risk factor for PDAC. This suggests that altered TRPV6 function may contribute to early pathogenic events before malignant transformation. While current evidence does not yet support TRPV6 as a standalone early detection marker, its integration into multi-marker genomic or transcriptomic panels may improve risk assessment and subtype classification. Taken together, current evidence suggests that TRPV6 is a clinically relevant Ca^2+^ channel in PDAC, with potential use as a prognostic biomarker, a regulator of therapy response, and a marker for future molecular stratification. However, larger patient cohorts and standardized detection methods are needed before TRPV6 can be implemented in routine clinical practice.

## 6. Therapeutic Targeting of TRPV6

TRPV6 inhibitors mainly include small-molecule compounds and peptide-based antagonists. In recent years, antibody-based approaches have also emerged, and several non-selective pharmacological modulators have been described. These antagonists mainly differ in their specificity and translational potential (Table 2).

### 6.1. Small Molecule Inhibitors

#### 6.1.1. TH-1177

TH-1177 is one of the earliest small-molecule inhibitors of TRPV6. Previous studies demonstrated that TH-1177 inhibits TRPV6-mediated Ca^2+^ uptake in prostate cancer cell lines [85]. Moreover, TH-1177 significantly reduced tumor growth and prolonged survival in prostate cancer xenograft models, suggesting that it may represent a potential therapeutic target in this type of carcinoma [86]. Despite these promising preclinical findings, TH-1177 remains an early-stage compound with only moderate selectivity for TRPV6 [85,87].

#### 6.1.2. Cis-22a

Cis-22a is one of the most potent and selective inhibitors of TRPV6. This compound blocks TRPV6-mediated Ca^2+^ currents even in the nanomolar concentration range, with an IC_50_ of ~82 nM in human TRPV6-expressing cell lines [88]. Structural and functional analyses show that cis-22a binds within the intracellular part of the TRPV6 pore, where it stabilizes a non-conducting channel conformation, thereby effectively decreases the Ca^2+^ permeability of the channel. The binding site of cis-22a partially overlaps with the CaM interaction site, therefore functionally mimics CaM-mediated channel inactivation [89]. The high selectivity of cis-22a for TRPV6 was confirmed by mutational analyses in which the amino acid substitutions within the pore region strongly reduced the inhibitory effect of the drug [88]. Despite its high potency and selectivity, cis-22a has not yet been tested in clinical settings, as further optimization is required to address limitations of the drug.

#### 6.1.3. Tetrahydrocannabivarin

Tetrahydrocannabivarin (THCV) is a natural derivative extracted from Cannabis sativa. This compound inhibits channel activity through a different mechanism than cis-22a. THCV binds to a unique membrane-accessible portal site, which revealed a previously unrecognized part of the channel [90]. Upon binding, THCV induces conformational changes in the transmembrane region that alter channel gating and stabilize the closed state. This process does not affect the structure of the selectivity filter. Electrophysiological recordings and Ca^2+^ imaging experiments have demonstrated that THCV, at micromolar concentrations, significantly reduces Ca^2+^ influx [90]. In a two-phase, dose-ranging, placebo-controlled trial, the adverse effects of THCV were investigated in healthy volunteers [91]. This clinical study showed that THCV has a favorable safety profile. Most of the side effects were mild, and the most common was euphoric mood. These results indicate that the drug is well tolerated and does not cause serious adverse effects. These favorable pharmacological properties of THCV suggest that this compound could be a potential drug candidate for the treatment of PDAC.

### 6.2. Peptide Inhibitors

#### SOR-C13 and SOR-C27

SOR-C13 and SOR-C27 are the most investigated peptide inhibitors of TRPV6. Both compounds are derived from the C-terminal region of the peptide, soricidin, which was originally isolated from the saliva of the northern short-tailed shrew (Blarina brevicauda). These peptides display high affinity and selectivity for TRPV6, with inhibitory concentrations in the low-nanomolar range. Due to these favorable properties, SOR peptides are considerably more potent inhibitors of the TRPV6 channel than small-molecule inhibitors [84,92]. Both SOR-C13 and SOR-C27 bind to an extracellular pocket of TRPV6 and stabilize the channel in a non-conducting state, ref. [92], that strongly decreases the Ca^2+^ permeability of the channel. SOR-C13 and SOR-C27 exhibit IC_50_ values of 14 and 65 nM, respectively, which are considerably lower than those of small-molecule inhibitors [92]. Electrophysiological recordings have shown that both peptides bind to the channel in its open state with slow dissociation kinetics [92]. Moreover, in mouse xenograft models of ovarian and prostate cancer, SOR-C27 was conjugated to superparamagnetic iron oxide (SPIO) contrast particles (SPIO-SorC27), enabling MRI visualization of its distribution and accumulation at tumor sites, which also reflected the high expression of TRPV6 in these tumors [92]. These results suggest that SOR peptides could potentially be used to deliver chemotherapeutic agents to TRPV6-expressing tumors and may therefore have potential applications in the diagnosis and treatment of various cancers. The anti-tumor activity of these peptides has also been demonstrated in a xenograft model of ovarian cancer [19]. Intraperitoneal administration of SOR-C13 at doses of 400, 600, and 800 mg/kg for 12 days inhibited tumor growth by up to 59% at the highest dose compared with untreated controls. Similarly, SOR-C27 at 800 mg/kg reduced tumor growth by 55% after 12 days. These results indicate that both peptides represent promising drug candidates for cancer therapy. This notion is supported by the finding that the anti-tumor activity of SOR-C13 has been demonstrated in a Phase I clinical study in patients with advanced tumors [84]. Intravenous administration of the drug at 6.2 mg/kg proved to be safe and well-tolerated, with no signs of toxicity. The drug stabilized the disease in 55% of patients, and in one patient with PC, a 27% reduction in tumor size was observed, which was associated with a significant decrease in CA19-9 levels. These findings suggest that SOR-C13 may represent a potential anticancer agent and justify further clinical investigation of the drug.

### 6.3. Antibody-Based Targeting of TRPV6

Earlier studies demonstrated that antibodies targeting extracellular epitopes of TRPV6, such as rb79 and rb82, can induce a biphasic channel response characterized by transient TRPV6 activation and enhanced Ca^2+^ entry. The transient TRPV6 activation–associated increase in Ca^2+^ influx leads to cell death in TRPV6-expressing prostate tumor cells, whereas this effect is not observed in TRPV6-deficient cells [93]. Building on these findings, a recent study described a novel TRPV6-targeting monoclonal antibody (mAb82) that binds to the extracellular region of the channel pore, corresponding to the same epitope previously used to generate the rabbit polyclonal antibody rb82 [83,93]. mAb82 inhibits Ca^2+^ influx in a dose-dependent manner and significantly reduces cell survival in prostate cancer cell lines by inducing apoptosis. The beneficial effects of mAb82 were also confirmed in xenograft mouse models, where the treatment markedly reduced tumor size, in some cases resulting in up to a 90% reduction in tumor growth, and significantly improved animal survival. Since the antibody did not cause significant toxicity in vivo, these findings suggest that mAb82 may represent a promising therapeutic strategy for the treatment of TRPV6-expressing tumors.

### 6.4. Non-Selective Modulators of TRPV6 Activity

In addition to selective TRPV6 inhibitors, several pharmacological agents have been reported to inhibit TRPV6-mediated Ca^2+^ entry; however, most of these compounds lack specificity and inhibit other Ca^2+^-permeable channels as well [102,103]. Classical trivalent cations such as Gd^3+^ and La^3+^ inhibit TRPV6 currents, but these compounds are also non-selective blockers of various Ca^2+^-permeable channels, such as CRAC and other TRP channels [34,94,95,96,97]. Ruthenium red, which is a polyvalent cationic dye, is also able to suppress TRPV6 activity but is not selective for TRPV6 and inhibits other TRPV and cation channels [98,99]. Similarly, 2-APB and GSK compounds affect multiple channel families, including ORAI, TRPC, and TRPV members [100,101]. Azole antifungals, such as econazole, have been reported to modulate TRP channel activity and Ca^2+^ influx, but their effects are not selective for TRPV6 and likely involve membrane perturbation and cytochrome P450–related mechanisms [99]. Collectively, these agents are best regarded as experimental tools for probing Ca^2+^-dependent processes rather than selective TRPV6 antagonists with clear translational potential.

## 7. Future Perspectives for TRPV6 in Pancreatic Ductal Adenocarcinoma

Accumulating evidence suggests that TRPV6 not only regulates Ca^2+^ homeostasis in cells but also contributes to tumor cell proliferation, survival, and resistance to apoptosis. Overexpression of TRPV6 has been reported in several cancers, including PC, suggesting that dysregulated Ca^2+^ influx plays a role in tumor progression. Therefore, several efforts have been directed toward the development of selective TRPV6 inhibitors. These compounds have been shown to effectively reduce tumor growth in various experimental models, and the most promising inhibitor, SOR-C13, has already advanced to Phase I clinical trials. At the same time, precise regulation of TRPV6 activity is critical for tumor cell survival, since both inhibition and excessive activation of the channel can reduce tumor growth. This observation suggests that TRPV6 functions more as a “Ca^2+^ gatekeeper” rather than a simple regulator of Ca^2+^ entry. Therefore, therapeutic strategies may need to move beyond complete channel blockade toward more refined approaches, such as partial or state-dependent modulation. However, it is important to consider that TRPV6 plays a fundamental role in physiological Ca^2+^ absorption in epithelial tissues, particularly in the intestine and kidney, where it contributes to systemic Ca^2+^ homeostasis [26,27]. Therefore, systemic inhibition of TRPV6 may lead to adverse effects such as impaired Ca^2+^ absorption, hypocalcemia, and secondary alterations in bone metabolism. Clinical observations from early-phase studies with the peptide inhibitor SOR-C13 support this concern, as transient treatment-related hypocalcemia was reported in several patients during dose-escalation trials [84]. Although Ca^2+^ and vitamin D supplementation reduced the severity of these events, these findings suggest that long-term TRPV6 inhibition may require careful monitoring of systemic Ca^2+^ balance [84]. Another important challenge is that TRPV6 is not exclusively expressed in tumor tissues, which may limit the therapeutic window of systemic TRPV6 blockade [29]. These potential toxicities represent a critical challenge for the clinical translation of TRPV6-targeted therapies. To overcome these limitations, future strategies may need to focus on tumor-selective targeting approaches, such as antibody-based delivery systems, local inhibition, or context-dependent modulation of channel activity, in order to minimize systemic side effects while preserving therapeutic efficacy. Nevertheless, the development of more effective therapies may greatly benefit from experimental models, including patient-derived organoids and genetically engineered mouse models, which may help to better understand the role of TRPV6 at different stages of disease progression. Taken together, TRPV6 may represent an important component of future anticancer strategies in PC; however, further efforts are needed to develop highly specific compounds with favorable pharmacokinetic and toxicological properties that are capable of effectively targeting this aggressive disease.

## 8. Limitations of Current Knowledge and Review

Despite increasing interest in TRPV6 as a regulator of Ca^2+^ signaling in pancreatic diseases and cancer, several important limitations should be acknowledged. First, direct evidence regarding the physiological expression and function of TRPV6 in normal pancreatic ductal cells remains limited, and many conclusions are based on indirect observations or pathological conditions. In addition, a substantial proportion of the mechanistic insights discussed in this review are derived from experimental studies performed in non-PC models or in vitro systems, which may not fully reflect the complexity of PDAC biology in vivo. Another limitation is the relatively small number of studies specifically investigating TRPV6 in human PDAC tissues and clinically relevant patient-derived models. An additional important limitation is the lack of data regarding TRPV6 expression and function across molecularly distinct PDAC subtypes, including classical and basal-like tumors, which differ substantially in their transcriptional programs, aggressiveness, and therapeutic responses. Similarly, the potential association between TRPV6-mediated Ca^2+^ signaling and common PDAC driver mutations, such as KRAS, TP53, CDKN2A, and SMAD4 alterations, remains poorly understood. Since these genetic and molecular alterations strongly influence PDAC biology and treatment response, clarifying their relationship with TRPV6 may be important for future patient stratification and therapeutic targeting. Finally, although several TRPV6-targeting compounds have shown promising preclinical activity, clinical data regarding their long-term efficacy and systemic safety are still limited. Taken together, these limitations highlight the need for further mechanistic, translational, and clinical studies to better define the role of TRPV6 in PDAC progression and therapeutic targeting.

## 9. Conclusions

TRPV6 is a crucial factor in regulating the balance of Ca^2+^ within cells and is linked to many different signaling pathways contributing to PDAC development such as NFAT, PI3K/AKT/mTOR, and NF-κB. Dysregulation of TRPV6-mediated Ca^2+^ influx promotes tumor cell proliferation, survival, metabolic adaptation, inflammatory signaling, invasion, and drug resistance. TRPV6 is also implicated in normal Ca^2+^ regulation in epithelial tissue aside from its pathological role in PC development. Recent advances in the understanding of TRPV6 structure and signaling mechanisms have facilitated the development of selective pharmacological inhibitors, peptide antagonists, and antibody-based targeting strategies. Some of these substances were found to show promising antitumor effects during animal testing, whereas SOR-C13 is currently undergoing early-stage clinical trials. Nevertheless, important limitations remain, including the incomplete understanding of TRPV6 biology in PDAC subtypes, the limited availability of clinically relevant human data, and the potential systemic consequences of long-term TRPV6 inhibition.

Taken together, current evidence indicates that TRPV6 represents a promising therapeutic target and potential biomarker in PDAC. However, further mechanistic, translational, and clinical studies are required to better define its role in pancreatic tumor biology and to support the development of safe and effective TRPV6-targeted therapies.

## Figures and Tables

**Table 1:** Cell-Type–Specific Expression and Function of TRPV6 in the Pancreas.

Cell Type	TRPV6 Expression	Putative Function	Pathophysiological Relevance	References
Ductal cell	Not clearly demonstrated, but transcriptomic data indicate its presence in the exocrine pancreas	It is hypothesized to regulate luminal Ca^2+^ levels by mediating Ca^2+^ uptake from the ductal fluid	TRPV6 loss-of-function → altered Ca^2+^ signaling and increased risk of chronic pancreatitis. Upregulation in pancreatic cancer	[22,24,36,37,38]
Acinar cell	Expressed and functionally active	Replenishment of intracellular Ca^2+^ stores during increased secretory activity	Pathological Ca^2+^ overload → mitochondrial dysfunction, premature enzyme activation, and acute pancreatitis	[23,39]
Endocrine pancreas	Transcriptomic studies indicate TRPV6 expression in both β- and α-cells	Proliferation and gene transcription	Reduced TRPV6 → decreased β-cell proliferation and insulin mRNA expression. Potential role in neuroendocrine tumor proliferation	[40,41,42,43]

**Table 2:** Classification, Selectivity, and Experimental and Clinical Relevance of TRPV6 Inhibitors.

Type	Inhibitor	Selectivity	Experimental/Clinical Relevance	References
Small-molecule inhibitor	TH-1177	Moderate	Reduced tumor growth and prolonged survival in prostate cancer xenograft models	[85,86,87]
	Cis-22a	High	Blocks TRPV6-mediated Ca^2+^ currents (IC_50_ of ~82 nM) in human TRPV6-expressing cell lines	[88,89]
	Tetrahydrocannabivarin	Moderate	Favorable safety profile in clinical trial; potential candidate for PDAC therapy	[90,91]
Peptide inhibitor	SOR-C13	High	Strong antitumor activity; Phase I trial showed good tolerability and disease stabilization	[19,84,92]
	SOR-C27	High	Can be used for tumor imaging and experimental cancer models	
Monoclonal antibody	mAb82	High	Induces apoptosis and reduces tumor growth in prostate cancer xenograft models	[83,93]
Non-selective inhibitor	Gd^3+^, La^3+^	Low	Inorganic inhibitor. Also blocks CRAC and other TRP channels	[34,94,95,96,97]
	Ruthenium red	Low	Polyvalent cationic dye. Widely used experimental Ca^2+^ channel inhibitor	[98,99]
	2-APB, GSK compounds	Low	SOCE/TRP modulator	[100,101]
	Econazole	Low	Azole antifungal and TRP modulator	[99]

## Data Availability

No new datasets were generated or analyzed during the current study. All information discussed in this review is derived from previously published studies, which are cited in the manuscript.
